# Metastatic Carcinomatosis Cirrhosis: A Rare Pattern of Metastasis

**DOI:** 10.7759/cureus.3876

**Published:** 2019-01-13

**Authors:** Phillip Knouse, Christie Hancock, Suzanne Iwaz, Pamela Kaiser

**Affiliations:** 1 Internal Medicine, Advocate Lutheran General Hospital, Park Ridge, USA; 2 Pathology, University of Illinois, Chicago, USA

**Keywords:** breast cancer, metastatic disease, liver failure, liver cirrhosis, liver metastases, hepatic cirrhosis, hepatic tumor

## Abstract

Metastatic carcinomatosis cirrhosis is a pattern of metastasis in which malignancy infiltrates the liver and provokes hepatic fibrosis. It is an especially rare complication of several malignancies, including breast cancer. We report a case of a 61-year-old woman with lobular carcinoma of the breast who presented with confusion and rising serum tumor markers without evidence of disease recurrence on imaging. She subsequently developed clinical evidence of hepatic dysfunction and a liver biopsy revealed diffuse infiltration of the liver by breast carcinoma with surrounding fibrous tissue deposition, consistent with metastatic carcinomatosis cirrhosis. This case highlights a rare and clinically significant pattern of metastasis and is the first to describe lobular carcinoma of the breast causing metastatic carcinomatosis cirrhosis.

## Introduction

Metastatic carcinomatosis cirrhosis is a rare condition described in several malignancies in which the liver is diffusely infiltrated by metastases [1-3]. Patients with this disease present with signs and symptoms of acute liver failure and frequently follow an aggressive clinical course leading to death [3]. Unlike discrete hepatic mass lesions, metastatic carcinomatosis cirrhosis may not be detectable with imaging and, often, biopsy or autopsy is required to confirm the diagnosis [3].

## Case presentation

**Figure 1:**
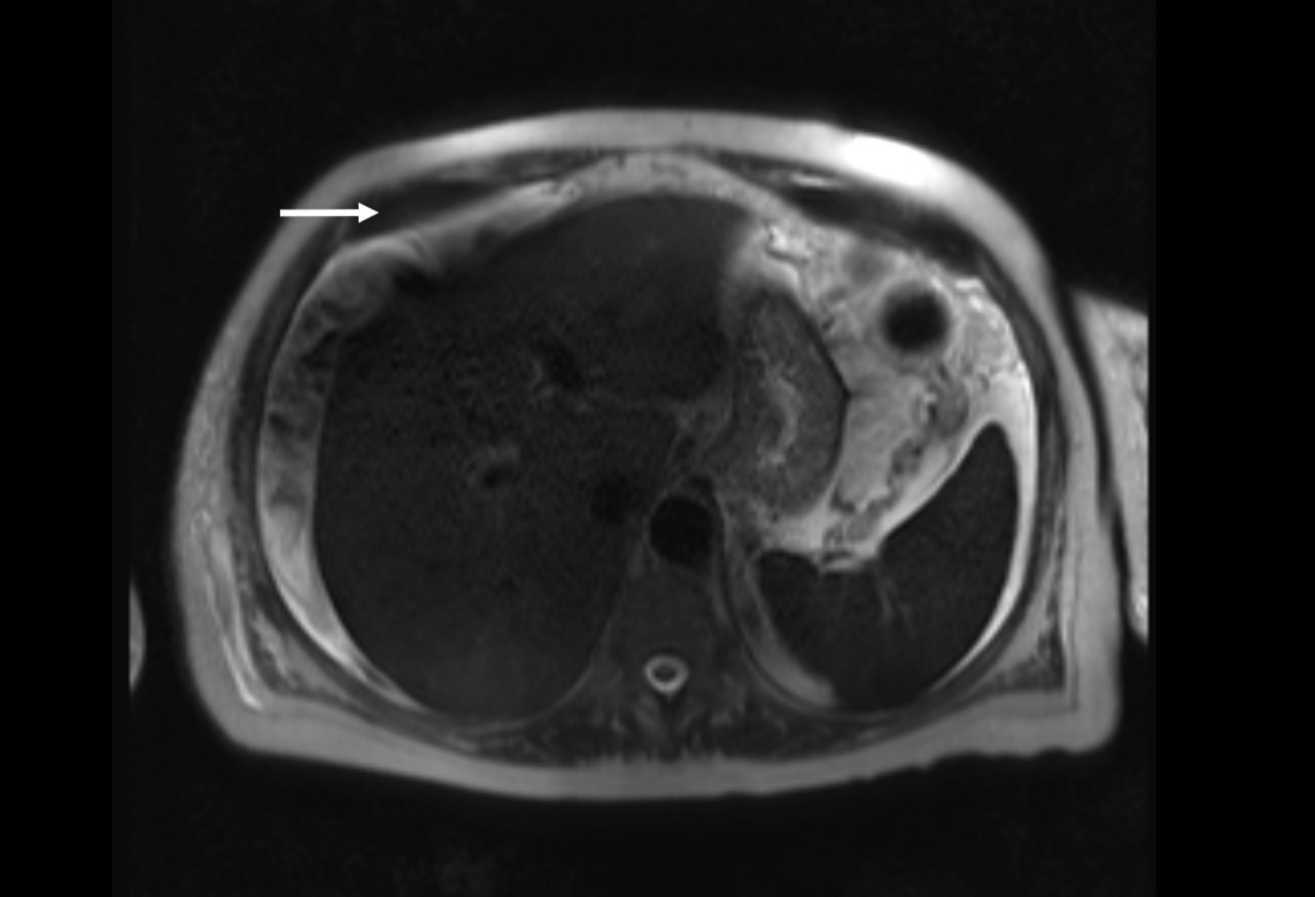
Abdominal MRI Normal appearing liver with a moderate volume of ascites

Here, we present a 61-year-old woman who presented to a community hospital with two weeks of progressive confusion and generalized weakness. She had been diagnosed four years earlier with stage IIIA, estrogen receptor-positive, progesterone receptor-positive, human epidermal growth factor receptor 2/neu non-amplified infiltrating lobular carcinoma of the breast. She underwent mastectomy and adjuvant therapy with adriamycin and cyclophosphamide, followed by paclitaxel, radiation, and anastrozole. Three years after her diagnosis, she developed an isolated bone metastasis for which she received local radiation, exemestane, and everolimus. The patient was unable to tolerate everolimus and her therapy was changed to palbociclib and fulvestrant. She was maintained on this regimen for more than one year, with no evidence of disease recurrence. However, in the months leading up to her hospitalization, her serum levels of cancer antigen (27.29 U/mL) and carcinoembryonic antigen began to rise. Despite an increase in these tumor markers, there was no evidence of disease recurrence on physical exam or imaging, including bone scan, computed tomography, and positron emission tomography. The lab results at that time showed hypercalcemia (corrected calcium 11.9 mg/dL), transaminitis (aspartate aminotransferase (AST) 88 U/L, alanine aminotransferase (ALT) 45 U/L), and hyperbilirubinemia (total bilirubin 1.8 mg/dL). Magnetic resonance imaging (MRI) of the brain revealed no abnormalities. Additional workup revealed parathyroid hormone-independent hypercalcemia and an elevated ammonia level (95 µmol/L). The patient's hypercalcemia was attributed to a paraneoplastic syndrome and she was started on gemcitabine. An abdominal MRI revealed a normal appearing liver with a moderate volume of ascites (Figure 1). Cytology of the ascites fluid confirmed a metastatic adenocarcinoma consistent with her known history of breast cancer.

**Figure 2:**
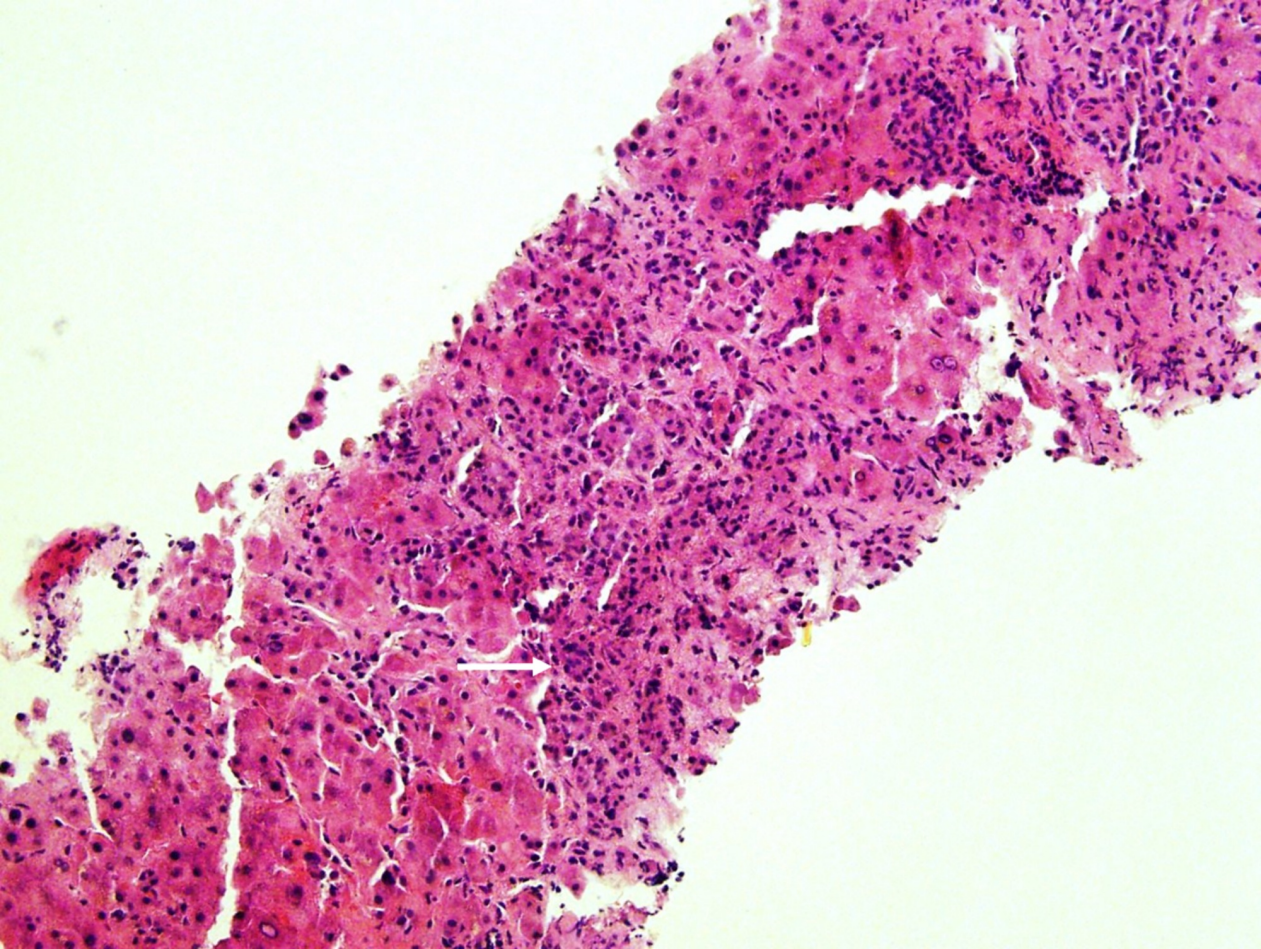
Liver Biopsy, Hematoxylin and Eosin Stain Atypical tumor cells with extensive fibrosis and desmoplastic reaction

**Figure 3:**
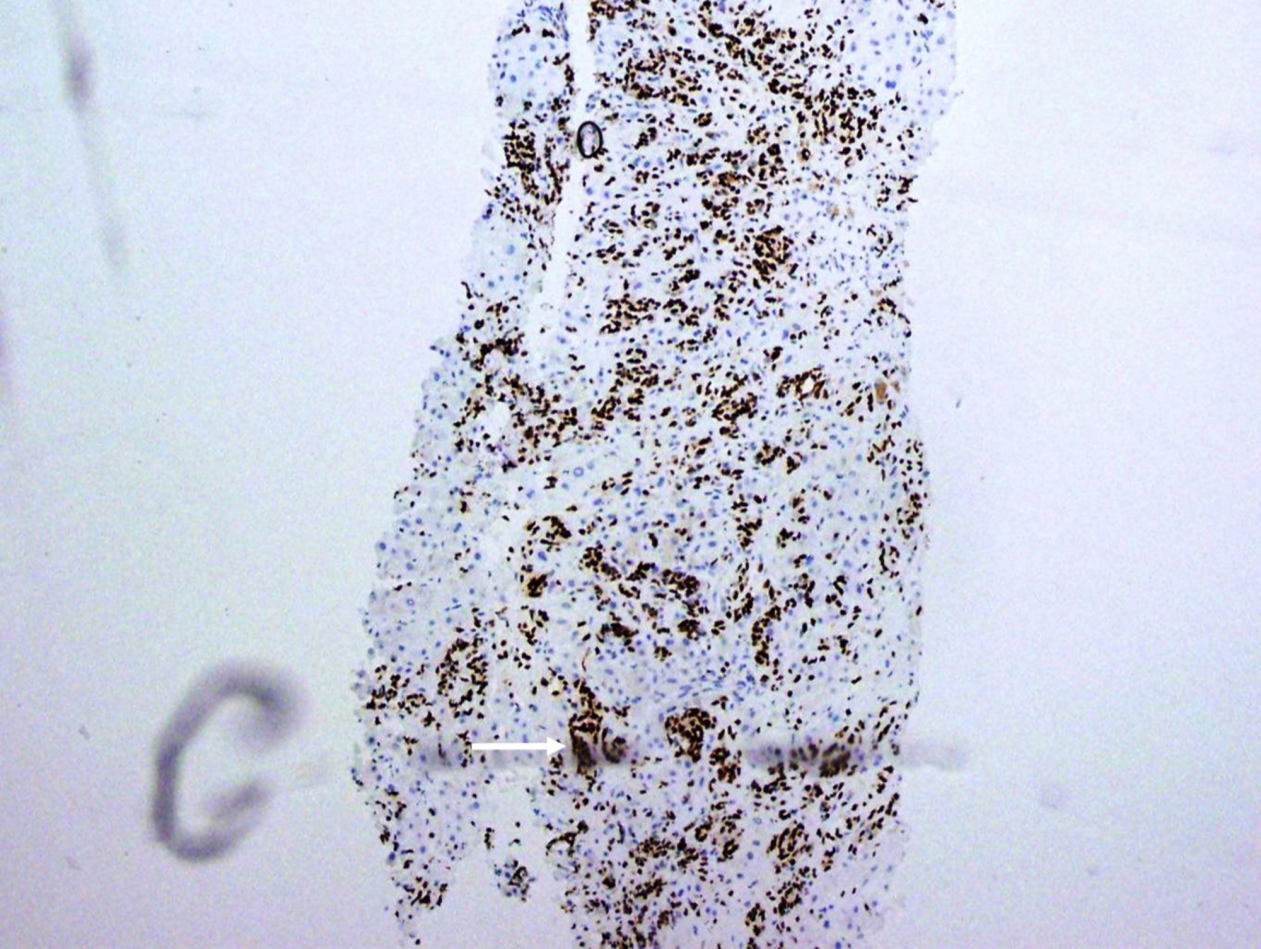
Liver Biopsy, GATA3 Stain Tumor cells with strong reactivity for GATA3, a highly breast-specific stain

**Figure 4:**
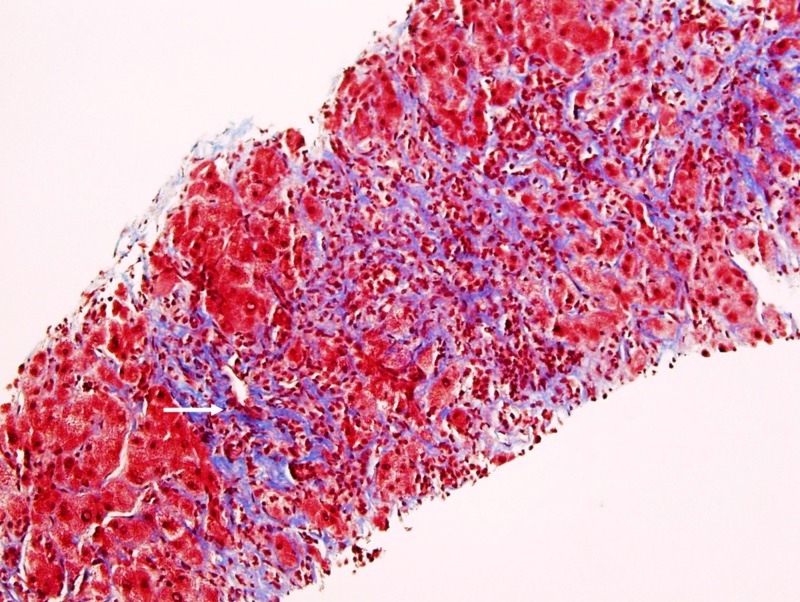
Liver Biopsy, Trichrome Stain Marked bridging fibrosis present adjacent to the tumor cells

The patient continued gemcitabine and her mental status, hypercalcemia, and ammonia level improved. However, she returned to the hospital two months later with jaundice, abdominal distention, and worsening encephalopathy. The lab reports were notable for recurrent hypercalcemia (corrected calcium 12.4 mg/dL), transaminitis (AST 86 U/L, ALT 54 U/L), coagulopathy (international normalized ratio 2.3), and hyperbilirubinemia (16.7 mg/dL). A transjugular liver biopsy revealed an elevated hepatic-portal venous pressure gradient, and histologic analysis confirmed metastatic breast carcinoma infiltrating the portal tracts and associated pericellular bridging fibrosis (Figures 2-4), a finding consistent with metastatic carcinomatosis cirrhosis. The patient enrolled in hospice and died shortly after.

## Discussion

The liver is a frequent site of metastasis but metastatic disease rarely results in acute liver failure [1-2]. Hepatic metastases are typically discrete mass lesions, whereas metastatic carcinomatosis cirrhosis is characterized by the diffuse infiltration of the liver by malignancy. Watson et al. first reported metastatic carcinomatosis cirrhosis in 1955 when describing cases of disseminated bronchogenic carcinoma causing fulminant liver failure [4]. More recently, the disease has been reported with several other tumor types, including colon, pancreas, lung, stomach, kidney, adrenals, and lymphomas [2-6]. Thus far, all reported cases of breast cancer causing metastatic carcinomatosis cirrhosis have been due to disseminated ductal carcinoma of the breast or to a not otherwise specified subtype of breast cancer. To our knowledge, this is the first reported case of a lobular carcinoma type of the disease.

The clinical presentation of metastatic carcinomatosis cirrhosis varies but some commonalities can be drawn from published case reports. Women in cases reported thus far are typically middle-aged, with a median age of 47 in one review published by Allison et al. [3]. Patients frequently experience a symptom-free interval after treatment of the primary malignancy before they develop nonspecific symptoms, including weight loss, nausea, and abdominal pain, consistent with our case. Patients ultimately develop hepatic dysfunction and evidence of liver failure such as bleeding varices, accumulation of ascites, jaundice, and hepatic encephalopathy [2-3,5].

Diagnosing metastatic carcinomatosis cirrhosis is difficult without a high index of suspicion, as imaging studies may not reveal metastatic infiltration of the liver or characteristic cirrhotic findings, as we observed with our patient [3,6]. When imaging is indicative of a hepatic process, it can show capsular retraction, a decrease in liver size, and evidence of portal hypertension such that the terms metastatic carcinomatosis and pseudocirrhosis are occasionally used interchangeably [7-9]. Diagnosis is also complicated by the fact that subtle hepatic impairment can be attributed to other causes. In this patient, a remote history of alcohol use was confounding. Definitive diagnosis of metastatic carcinomatosis cirrhosis requires a transjugular biopsy or postmortem autopsy with histologic findings of diffuse intrasinusoidal infiltration of the liver resulting in extensive fibrous tissue deposition [10].

Patients with metastatic carcinomatosis cirrhosis have a dismal prognosis, and there is no consensus on how best to manage these patients [2,11]. Treatment options are limited because active malignancy is a contraindication to liver transplantation and hepatic impairment limits chemotherapy options [2-3]. Expectedly, in the review by Allison et al., 18 of 21 patients presented with acute liver failure and the median survival of these patients was three weeks [3].

## Conclusions

In conclusion, we suggest that metastatic carcinomatosis cirrhosis should be considered in patients with a history of malignancy who present with laboratory or clinical evidence of hepatic dysfunction. This case establishes that, as with other types of malignancy, disseminated lobular carcinoma of the breast may cause this pattern of metastasis.
